# Do Herbal Supplements and Probiotics Complement Antibiotics and Diet in the Management of SIBO? A Randomized Clinical Trial

**DOI:** 10.3390/nu16071083

**Published:** 2024-04-07

**Authors:** Lucia Redondo-Cuevas, Lucia Belloch, Vanesa Martín-Carbonell, Angela Nicolás, Iulia Alexandra, Laura Sanchis, Marina Ynfante, Michel Colmenares, María Mora, Ana Reyes Liebana, Beatriz Antequera, Francisco Grau, José Ramón Molés, Rubén Cuesta, Samuel Díaz, Noelia Sancho, Héctor Tomás, José Gonzalvo, Mercedes Jaén, Eva Sánchez, Ana Garayoa, Nadia Moreno, Ana Gallén, Ernesto Cortés-Castell, Xavier Cortés-Rizo

**Affiliations:** 1 Valencian Digestive Institute (IVADI), 46021 Valencia, Spain; lucia@redondocuevas.es (L.R.-C.); luciabellochperez@hotmail.com (L.B.); ynfafer@hotmail.com (M.Y.); michelcobul6@gmail.com (M.C.); anareyesliebana@gmail.com (A.R.L.); fgraug@gmail.com (F.G.); jrmolesmarco@gmail.com (J.R.M.); noeliasanchopsic@gmail.com (N.S.); mercedes.jaen.revuelta@gmail.com (M.J.); xacori@gmail.com (X.C.-R.); 2 Digestive Section, Hospital de Sagunto Internal Medicine Service, 46520 Valencia, Spain; iulia.s.nutricion@gmail.com (I.A.);; 3 Department of Pharmacology, Pediatrics and Organic Chemistry, Miguel Hernández University of Elche, 03550 Elche, Spain; ernesto.cortes@umh.es

**Keywords:** Small Intestine Bacterial Overgrowth (SIBO), herbal therapies, dietary supplements, antibiotics, probiotics, FODMAP diet

## Abstract

Small intestinal bacterial overgrowth (SIBO) arises from dysbiosis in the small intestine, manifesting with abdominal symptoms. This study aims to assess the efficacy of combined antibiotic therapy, herbal supplements, probiotics, and dietary modifications in SIBO management. A total of 179 SIBO-diagnosed patients underwent clinical evaluation and breath testing. Patients were categorized into hydrogen (H_2_-SIBO) and methane (CH_4_-SIBO) groups. The control group received standard antibiotic therapy and a low-FODMAP diet, while the intervention group received additional herbal antibiotics, probiotics, and prebiotics. After treatment, both groups exhibited reduced gas levels, particularly in CH_4_-SIBO. Clinical remission rates were higher in the intervention group, especially in CH_4_-SIBO cases. Logistic regression analysis showed gas concentrations at diagnosis as significant predictors of treatment success. In conclusion, adjunctive herbal supplements and probiotics did not significantly impact gas levels, but showed potential for clinical improvement, especially in CH_4_-SIBO.

## 1. Introduction

The microbiota is the set of microbes that colonize a large part of our bodies, including the digestive tract, the skin, or the genital tract, in a symbiotic relationship with the host, playing a vital role in digestive, defensive, metabolic, trophic, and endocrine functions. When the microbiota is diverse and resilient, the organism is in eubiosis. However, due to the influence of different factors, this balance may become disrupted, and the entire ecosystem chain starts to fail, resulting in an imbalance of the microbiota called dysbiosis [1,2].

This imbalance of the microbiota can have negative health consequences. One of them, related to dysbiosis in the small intestine, is small intestinal bacterial overgrowth (SIBO) [3,4]. This is a very prevalent condition in patients with irritable bowel syndrome (IBS) [5].

SIBO usually presents with symptoms of abdominal bloating and distension, flatulence, abdominal pain, and altered bowel habits. It also often produces extraintestinal symptoms, including asthenia or headache, as well as cardiovascular, endocrine, neurological, neurological, nephrological, connective tissue, or dermatological complications. Among other mechanisms, it has been described that SIBO is associated with an increase in lipopolysaccharides and inflammatory cytokines in the intestinal mucosa, which can cause intestinal hyper-permeability and contribute to chronic low-grade systemic inflammation [6], generating alterations beyond the digestive tract. 

SIBO is multifactorial in origin, with its most frequent causes being dysfunctional movement of contents through the small intestine (due to drugs such as antidepressants or intestinal stenosis or other anatomical alterations), reduced gastric acid secretion (due to gastric bypass or chronic use of proton pump inhibitors), reflux of colon contents into the small intestine due to ileocecal valve dysfunction, or due to causes that generate intestinal dysbiosis such as the use of antibiotics, poor diet with abuse of ultra-processed products, as well as poor emotional management (stress, anxiety, depression) [7,8].

Several SIBO types can be identified according to the predominant gas excreted by the microorganisms that produce it: hydrogen (H_2_-SIBO), methane (CH_4_-SIBO), and hydrogen sulfide. These are not mutually exclusive, and patients may present a combination, such as excessive H_2_ and CH_4_ gas production. 

Early studies regarding SIBO defined the diagnostic criteria for SIBO as a bacterial count greater than 10^5^ CFU/mL cultured from jejunal aspirate [4]. However, this is an invasive and rarely available technique. Glucose or lactulose breath tests have been validated to diagnose SIBO [9] and have enabled the diagnosis to be made upon clinical suspicion, which has resulted in a significant increase in the estimated incidence of SIBO in recent years. 

According to established guidelines, the standard treatment for the management of SIBO is antibiotics, although the dose and type is not well defined [10,11]. The most commonly used is rifaximin, a broad-spectrum antibiotic with low absorption and low bacterial resistance [12]. A systematic review and meta-analysis described that the use of rifaximin achieves a SIBO eradication rate of approximately 60% [13]. Other antibiotics such as metronidazole or neomycin are being used in association with rifaximin more in CH_4_-SIBO forms [10].

Other therapies have been emerging as aids in the therapeutic approach to SIBO, such as the use of medicinal plants (herbs). Supplementation with a mixture of plant extracts (oregano, berberine, wormwood, yarrow, thyme, ginger, licorice, etc.) has been described as being equally effective as rifaximin for the treatment of SIBO [14]. The same is true for supplementation with berberine alone [15]. More studies have evaluated the positive effects of herbal extracts for the clinical management of SIBO [16]. 

In addition, several studies have proposed the use of other aids to support the clinical management of SIBO. These include probiotics [17], glutamine [18,19,20], and partially hydrolyzed guar gum, a type of fiber with prebiotic actions that has been shown to drive improvement in IBS symptoms and stool consistency in patients with IBS-diarrhea [21].

In relation to diet, very little data exists on the best dietary strategy for patients with SIBO. Due to the similarities between the pathophysiology of IBS and SIBO, the use of diets low in fermentable polyols, monosaccharides, disaccharides, and oligosaccharides (FODMAPs) for the management of SIBO is widespread [22]. FODMAPs are a group of compounds that can produce gas through gut microbial fermentation and, in susceptible individuals, can worsen symptoms such as abdominal bloating and other gastrointestinal symptoms. The low FODMAP diet improves symptoms in 70% of IBS patients [23].

Although eradication of SIBO with antibiotics is considered the treatment of choice, in some cases it is not effective, and up to 43.7% of patients relapse within 9 months of completing antibiotic treatment [24]. Studies are therefore needed to identify other treatments for SIBO that focus on modifying the bacterial ecosystem, improving dysbiosis and quality of life.

The hypothesis of the present study is that the combined use of antibiotics together with herbal supplements with antibiotic properties, probiotics, glutamine, prebiotic fiber, and a low FODMAP diet could provide better results for the management of SIBO than the standard treatment consisting of antibiotics and a low FODMAP diet alone.

## 2. Materials and Methods

### 2.1. Study Population

A total of 179 patients diagnosed with SIBO were recruited based on the quantification of gases (hydrogen and methane) in exhaled breath. The patients attended the Gastroenterology Department of Sagunto Hospital (Valencia, Spain) and Casa de Salud Hospital (Valencia, Spain) from November 2021 to March 2023. 

Inclusion criteria were having a diagnosis of hydrogen or methane SIBO, adherence to the prescribed treatment for 3 months, and signing the informed consent form. Exclusion criteria included being under 18 years of age, having an associated chronic digestive disease (celiac disease, inflammatory bowel disease), intestinal parasites, elevated fecal calprotectin, and/or another possible cause of dysbiosis that could alter the results of the treatment, after evaluation by a gastroenterologist.

### 2.2. Data Collection

The results were collected from November 2021 to March 2023, and all patients were followed from the time of diagnosis through the next 3 months of treatment. 

#### 2.2.1. SIBO Diagnosis

Each patient performed a breath test at the beginning of the study and after 3 months. For the test, the patient ingested a substrate (lactulose or lactitol), and exhaled breath samples were collected every 25 min for 175 min. A graph showing the levels of hydrogen, methane, and carbon dioxide (CO_2_) was obtained with the results. The CO_2_ levels were evaluated to ensure proper sample collection. If hydrogen values rose 20 ppm from baseline before the 90th minute, the result was considered positive for H_2_-SIBO. If methane values rose above 10 ppm from baseline at any time on the graph, the result was considered positive for CH_4_-SIBO [25]. Mixed SIBO is defined when the patient presents positive H_2_-SIBO and CH_4_-SIBO in the same breath test.

#### 2.2.2. Complementary Analytical Tests

A routine blood test was performed at the beginning and end of treatment to determine the patient’s basic health values, as well as a stool culture to determine fecal calprotectin and the presence or absence of fecal parasites. The analytical test included examination of parameters such as vitamins B_12_, D, and folic acid, anti-transglutaminase antibodies, albumin, hemogram, cholesterol and triglycerides, and C-reactive protein.

#### 2.2.3. Measurement of Covariates

The variables age (years), sex, and BMI (kg/cm^2^) based on weight and height were collected. The participants were asked about smoking habits and whether they had a chronic disease, and if they had taken medication in the last 3 months. 

#### 2.2.4. Procedure

For inclusion in the study, patients had a consultation with a gastroenterologist and agreed to participate by signing the informed consent form. During the visit, the result of the breath test was recorded, and the diagnosis was confirmed. A medical history of the patient was taken, including anthropometry (weight, height), smoking, alcohol intake, and drug intake, and a blood and stool analysis was ordered. The physician prescribed treatment according to the type of SIBO. Subsequently, the patient attended a consultation with a dietitian-nutritionist who recorded the dietary habits of each patient to prescribe a low FODMAP diet tailored as much as possible to their needs and lifestyle, in order to achieve greater treatment adherence and satisfaction with the low FODMAP diet. Antibiotic and medicinal herb treatment was started together with the low FODMAP diet.

**Figure 1:**
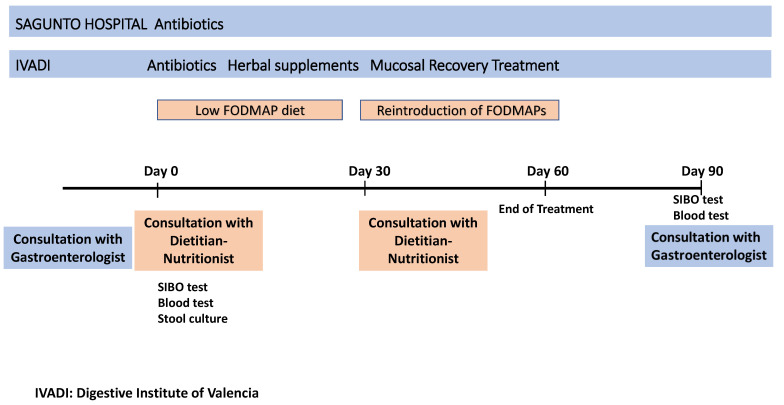
Diagram of the timeline of patient treatment and follow-up.

After 30 days of treatment, the patients returned for a follow-up consultation with the dietician-nutritionist, who explained how to reintroduce food during the next 8 weeks. At that time, a new breath test for SIBO and another blood test were ordered after 6 weeks of treatment (15 days before the fourth and final gastroenterologist visit). After 90 days of treatment, the patients had a final consultation with the gastroenterologist, in which the clinical symptoms and the results of the blood tests and SIBO breath test were reviewed (Figure 1).

#### 2.2.5. Treatment

With regard to treatment, the intervention group (IG) comprised patients receiving care at the Casa de Salud Hospital in Valencia, at the Digestive Institute of Valencia (IVADI). The treatment administered consisted of two phases and was prescribed to each patient according to the type of SIBO:

PHASE 1 cleansing (1 month):

- H_2_-SIBO: rifaximin 200 mg (2-2-2) for 10 days. After this, two commercial herbal preparations were recommended: Oleocaps 2 (Ingredients: rapeseed oil, gelatin, glycerin, essential oils (*Origanum vulgare*, *Cinnamomum cassia*, *Citrus limon*, *Mentha piperita*), extract rich in tocopherol) (Pranarom, Barcelona, Spain) 1-1-1, and berberine (Nutilab, Benaguasil, Valencia, Spain) 1-1-1, both for 20 days until completing the month. In patients with polypharmacy, wormwood (Nutri Holistic, Huelva, Spain) 2-2-2 was used instead of berberine. For gastritis, wormwood (Nutri Holistic) and berberine (Nutilab) were used as herbal supplements.
- CH_4_-SIBO: rifaximin 200 mg (2-2-2) for 10 days and neomycin 500 mg (1-0-1). If this caused symptoms, the dose was reduced to one tablet per day. During these 10 days, the patients took the probiotic Ultra Levura (*Saccharomyces boulardii*, 250 mg per day) (Laboratorios Salvat, SA., Esplugues de Llobregat, Spain). Subsequently, two commercial herbal preparations were recommended: Oleocaps 2 (Pranarom) 1-1-1 and wormwood (Nutri Holistic) 2-2-2. For gastritis, wormwood (Nutri Holistic) 2-2-2 and black cumin oil (Sura Vitasan, Renteria, Guipuzkoa, Spain) 1-1-1 were used.

In all cases a low FODMAP diet was initiated, using the Monash University food list (FODMAP app) [26].

PHASE 2 mucosal recovery (6 weeks):

- H_2_-SIBO: Probiotic Proinflam (Soria Natural, Garray, Soria, Spain) 1-0-0, L-glutamine (VByotics, Natural Probiotics, SL., Barcelona, Spain) 5 g/2 times daily between meals, and partially hydrolyzed guar gum (Max Fibra, Deiters, Badalona, Barcelona, Spain) 5 g daily, together with one of the meals.
- CH_4_-SIBO: Probiotic Proinflam (Soria Natural) 1-0-0 and L-glutamine (VByotics) 5 g/two times daily between meals.

Finally, the reintroduction of FODMAP foods was initiated.

The CG patients were given the same low FODMAP diet as the IG group, followed by subsequent FODMAP reintroduction, in addition to antibiotic treatment depending on the type of SIBO:

- H_2_-SIBO: rifaximin 200 mg (2-2-2) for 10 days.
- CH_4_-SIBO: rifaximin 200 mg (2-2-2) for 10 days and neomycin 500 mg (1-0-1). If this caused symptoms, the dose was reduced to one tablet per day.

### 2.3. Statistical Analysis

Quantitative variables were described through means and standard deviations, and qualitative variables through absolute (*n*) and relative (%) frequencies. To establish possible differences between initial and final values of the quantitative samples, a paired-sample Student’s *t*-test was performed, and for the qualitative variables, a Chi-square test was used. For comparison between the different SIBO subgroups, a Student’s *t*-test was used. Finally, to eliminate the influence of potential confounding variables, binary logistic regression analysis was carried out for all patients and by SIBO subgroups, with respect to whether exhaled gases normalized after 3 months of treatment. Statistical analyses were performed using the IBM SPSS v.26 statistical program (IBM Corp., Armonk, NY, USA). The significance level used was *p* < 0.05.

### 2.4. Ethical Considerations

The study was carried out in strict accordance with the international ethical recommendations for research and clinical trials in humans contained in the Declaration of Helsinki and was classified by the Spanish Agency of Medicines and Healthcare Products as a prospective follow-up post-authorization study on 12 February 2015 (FXC-TNF-2015-01). It was subsequently approved by the Clinical Research Ethics Committee of Sagunto Hospital on 22 July 2015 (FXC-TNF-2015-01) and endorsed by the local ethics committees of all the participating centers. Written informed consent was obtained from all the patients and study participants.

## 3. Results

The clinical course of 179 patients with SIBO undergoing treatment was followed for 3 months to assess whether exhaled gases and symptoms normalized and whether clinical improvement occurred. Of the patients included in the study, 56 were positive for H_2_-SIBO (24 CG and 32 IG) and 123 were positive for CH_4_-SIBO or both types of SIBO simultaneously (35 CG and 88 IG). The initial patient characteristics are described in the second column of Table 1, in which a predominance of women attending for consultation can be seen (82.7%). BMI was normal, and about one-fourth had a chronic non-digestive disease (26.9%). There were no significant differences in the main variables examined between the IGs and CGs. 

To assess whether there were differences in patient characteristics between the two types of SIBO, we compared their initial parameters with the values shown in the third and fourth columns of Table 1.

Clearly and by the same definition, the only variables showing differences between the two SIBO groups were the peak concentrations of exhaled H_2_ and CH_4._ When the frequency of excess exhaled gases was examined, methane excretors predominated (68.7%) compared to hydrogen excretors (31.3%). In the bivariate analysis, no differences were observed between CG and IG values in either type of SIBO, as shown in the results in Table 2.

In the same Table 2, when evaluating treatment success based on the decrease in exhaled gases, a clear reduction is found both in the CG and in the IG for CH_4_ (*p* ≤ 0.001 and 0.005, respectively). This is not as evident in the case of H_2_-SIBO, in which there was a decrease, but it was significant only in the IG (*p* = 0.006). However, no differences were observed in the reduction in gases in either type of SIBO when comparing the CG and the IG (*p* = 0.175 and *p* = 0.338). 

Nevertheless, when studying the number of patients whose exhaled gas values were normalized, only 74 patients (41.3%) had normal values, distributed according to treatment group and type of SIBO as shown in Table 3. It should be noted that there were no differences in success by type of treatment or type of SIBO in terms of the normalization of exhaled gases at the end of the trial.

When the results were analyzed according to clinical manifestations, no differences were found in the two CGs and IGs by type of SIBO. However, there was a significant difference (*p* = 0.038) in normalization of clinical manifestations between the CH_4_-SIBO CG (60.0%) and IG (78.4%) (Table 3).

To establish factors that may potentially influence the normalization of gas excretion in treated SIBO patients, the values of those who normalized (N) and those who did not (NN) were examined. These values are shown in Table 4.

**Figure 2:**
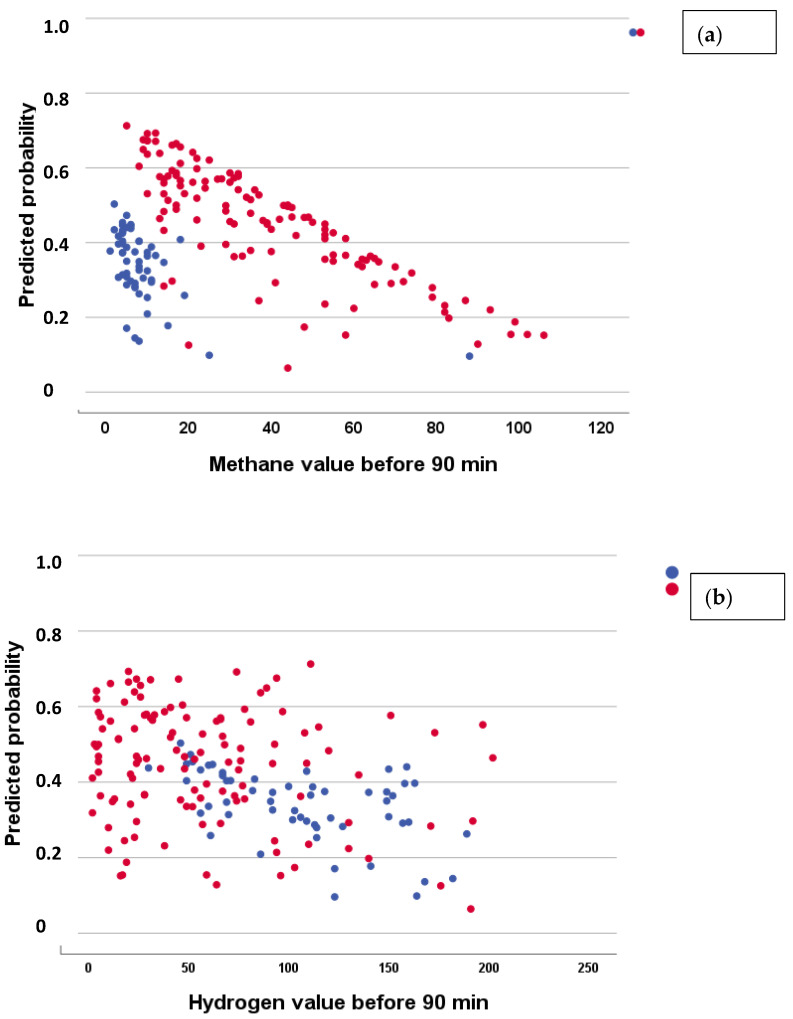
Diagnosed probability of normalization after treatment, according to the binary logistic regression model, in relation to the initial concentration of exhaled methane (**a**) and hydrogen (**b**). Red: CH_4_-SIBO; Blue: H_2_-SIBO.

Evaluation of these results in the two SIBO groups revealed that the only variables analyzed that could be influential factors in the success or failure of the treatment were patient age and the hydrogen and methane gases exhaled at diagnosis. The rest of the variables were far from significance. Accordingly, binary logistic regression analysis (normalization/non-normalization) was performed for all the variables studied using the stepwise method. A highly significant model was obtained (Chi-square = 16.288; *p* < 0.001) in which only the variable initial concentrations of the two gases remained, for CH_4_ (Exp B = 0.974, 95%CI 0.958–0.991; *p* = 0.020) and for H_2_ (Exp B = 0.986, 95%CI 0.974–0.998; *p* = 0.010), indicating that the probability of treatment success is lower with increasing concentrations and much higher for CH_4_-SIBO (Figure 2a) than for H_2_-SIBO (Figure 2b).

## 4. Discussion

SIBO is an increasingly common condition. However, the best treatment has not been clearly defined, and many patients relapse after treatment. Although natural treatments using herbal supplements and pre/probiotics are becoming increasingly popular for managing SIBO, to our knowledge, there are to date no similar publications comparing antibiotics following current clinical practice guidelines [10,11] with antibiotics combined with herbal supplements, probiotics, and other natural aids.

The present study compares two types of treatments for H_2_-SIBO and CH_4_-SIBO: one based on antibiotics and a low FODMAP diet (control group), and another using the same protocol, but with the addition of herbal supplements, probiotics, prebiotics, and glutamine (intervention group). Although the results showed no significant differences in the normalization of exhaled gas curves between groups, the patients in the IGs showed an improved response in gastrointestinal symptoms, especially in CH_4_-SIBO.

Our results show that, in general, SIBO is more frequent in women and the most common type of SIBO is CH_4_-SIBO, similar to what is reported in the literature [27,28,29]. 

According to the latest international consensus, antibiotics play a key role in the treatment of SIBO [10,11]. Many antibiotics have been evaluated for the management of SIBO, including amoxicillin with clavulanic acid, ciprofloxacin, doxycycline, metronidazole, neomycin, norfloxacin, tetracycline, cotrimoxazole, and rifaximin [30]. The goal of treatment is not to completely eradicate bacteria from the small intestine, but to modulate them in order to reduce SIBO symptoms. Currently, rifaximin is the antibiotic of choice for managing SIBO because of its good safety profile, placebo-like adverse event rates, low rate of resistance, and eubiotic effect through increasing beneficial bacterial strains [31]. Three recent meta-analyses evaluating the safety and efficacy of rifaximin in the treatment of SIBO demonstrated high successful eradication rates of around 70%) [32,33,34]. Our results are lower—about 40% resolution according to normalization of excreted gases, and over 70% according to clinical symptoms. There are several possible reasons for this. In principle, the dose of rifaximin used in our study was lower than that published in American studies: 1200 mg instead of 1650 mg per day, given that the baseline dosage of rifaximin in the USA is 550 mg and in Europe it is 200 mg per tablet. This could be one of the causes for finding lower rates of curve normalization in our study compared to others. Indeed, one study reported higher rates of curve normalization with rifaximin doses of 1600 vs. 1200 mg/day for 7 days [35]. Another potential reason for the differences observed in results is that the studies performed breath testing between 1 to 2 weeks after antibiotic completion, whereas in the present study this was performed 2.5 months after antibiotic completion, with normalization of the curve in the medium term. According to the literature, the further we move away from antibiotic administration, the worse the response rates are to the curve, with a relapse rate of over 40% [24]. In our study, curve normalization was higher in CH_4_-SIBO patients (44.7%) vs. H_2_-SIBO (33.9%) patients, without reaching significance. This could be due to the addition of neomycin to rifaximin in the treatment of CH_4_-SIBO [36]. Future lines of research should evaluate whether the addition of neomycin in H_2_-SIBO treatment could improve outcomes, as well as whether this improvement in the intervention group would be affected by the intake of probiotics (and prebiotics in the case of the H_2_-SIBO group) up to a week before the second breath test. Several studies have correlated probiotic intake with increased gas production [37] in breath tests, just as dietary fiber consumption (or prebiotic supplements) also increases exhaled gas [38].

It should be noted that, in our study, CH_4_-SIBO patients had a superior clinical response in the IG compared to the CG (78.4% vs. 60% *p* = 0.038). This could be explained by the addition of herbal supplements in this group of patients together with the prebiotic *Saccharomyces boulardii*, which has been associated with a reduction in gas excretion, mainly methane, leading to a significant improvement in patients with SIBO [39].

Finally, concerning the factors that can influence normalization in SIBO, both bivariate analysis and binary logistic regression analysis confirmed that the most important factor is the concentration of gases excreted at diagnosis; the higher the concentration, the worse the prognosis. Sex, smoking, chronic diseases, and obesity did not emerge as risk factors. In recent years there have been a large number of articles published on the risk factors for SIBO, showing great variability in the results [40].

The characteristic symptoms of SIBO are related to dysbiosis in the small intestine [38,41]. A treatment that aims to improve the gut microbiome can therefore more successfully improve the symptoms of SIBO. Herbal supplements are not only intended to control excess microbes, but also have effects on modulation of the gut microbiota and can also normalize gastrointestinal motility, relieve visceral hypersensitivity, modulate the hypothalamic-pituitary-adrenal axis, and control inflammation [42,43]. Probiotics have been used in numerous studies to successfully improve symptoms associated with SIBO [44]. Glutamine is an amino acid that can improve gut microbiota and intestinal barrier integrity through several mechanisms including reduction of the Firmicutes/Bacteroidetes ratio, control of pathogen colonization and overgrowth, increased production of secretory immunoglobulin A, and formation of tight junctions between enterocytes [18]. Several studies have reported that glutamine supplementation improves the symptoms associated with irritable bowel syndrome [19,20].

In the H_2_-SIBO group, a similar remission of symptoms was observed in the IG compared to the CG (71.9% vs. 70.8%, respectively), although the difference was not significant. These results may be due to the type of herbal supplements used, the addition of partially hydrolyzed guar gum (not used in the CH_4_-SIBO protocol), or because the probiotic *Saccharomyces boulardii* was included only in the CH_4_-SIBO treatment (not in H_2_-SIBO) during antibiotic treatment. 

### 4.1. Strengths and Limitations

A notable strength of this study is the confirmation of the factors influencing the normalization of breath test results by multivariate analysis, which eliminates potential cross-influences. Also of note is the homogeneity of the treatment followed in both the CGs and the IGs, given that the professionals were part of the same clinical team, and the treatments were agreed upon by all of the participants who conducted the study. Furthermore, the study patients corresponded to those usually seen in a general gastroenterology clinic with a diagnosis of SIBO, without focusing on a specific type of neurological, geriatric, or gastrointestinal disease that could be associated with the presence of SIBO. 

Concerning the limitations, we should mention the limited number of patients analyzed, especially in the H_2_-SIBO group. Nevertheless, almost all of the patients who sought care for this condition participated in the study. It is also important to note that there is no gold standard for a clear classification of normalization of either exhaled gas data or clinical symptoms, hence the wide discrepancy between the percentages of normalization of gases and of symptoms. This is a point that deserves further research effort.

### 4.2. Implications for Clinical Practice and Research 

Regarding future research, we believe that several areas should be explored in more depth. First, the eradication criteria for SIBO patients should be unified and, second, treatments should be further developed, including both antibiotic treatments and the use of herbal supplements and other natural treatments targeted at improving the intestinal microbiome (seeking diversity and resilience). In addition, the optimal type of diet for patients with SIBO should be examined, considering improvement of the gut microbiome, as well as patient adherence.

More research should be done on the mechanisms fostering improvement of clinical symptoms in patients with SIBO through treatment with a combination of antibiotics, herbal supplements, probiotics, and other natural treatments, as suggested by our results.

## 5. Conclusions

For the management of SIBO, the addition of herbal supplements and probiotics to standard treatment does not appear to improve results of the exhaled gas curve in the medium term. However, it could lead to significant clinical improvement in the medium term, especially in individuals with CH_4_-SIBO.

## Figures and Tables

**Table 1:** Description of the baseline characteristics of the two groups of H_2_-SIBO and CH_4_-SIBO patients and the differences between them.

Variable	Total (*n* = 179) Mean ± SD; *n* (%)	H_2_-SIBO (*n* = 56; 31.3%) Mean ± SD; *n* (%)	CH_4_-SIBO (*n* = 123; 69.7%) Mean ± SD; *n* (%)	*p*-Value
Age (years)	45.7 ± 16.2	46.4 ± 15.5	45.4 ± 16.5	0.347
Women	148 (82.7)	46 (82.1)	102 (82.9)	0.898
BMI (kg/m^2^)	23.9 ± 4.3	24.1 ± 4.0	23.9 ± 4.4	0.218
H_2_ (ppm) *	34.3 ± 31.6	45.6 ± 28.3	29.2 ± 31.8	<0.001
CH_4_ (ppm) **	29.6 ± 25.4	8.8 ± 11.7	39.1 ± 24.3	<0.001
Smokers	21 (11.7)	5 (8.9)	16 (13.0)	0.432
Chronically ill	40 (22.3)	11 (19.6)	29 (23.6)	0.597

* Maximum value before 90^′^ in the SIBO breath test. ** Maximum value at any time of the SIBO breath test.

**Table 2:** Comparison between exhaled gases at diagnosis and after 3 months of treatment, by CG and IG in both types of SIBO analyzed.

	H_2_-SIBO H_2_ (ppm) Mean ± SD			CH_4_-SIBO CH_4_ (ppm) Mean ± SD		
	Control *n* = 24	Intervention *n* = 32	*p*-Value	Control *n* = 35	Intervention *n* = 88	*p*-Value
Diagnosis	47.0 ± 30.3	44.6 ± 27.2	0.377	37.6 ± 24.1	39.7 ± 24.5	0.340
3 months	31.9 ± 34.0	38.3 ± 33.7	0.242	28.0 ± 32.8	25.7 ± 31.9	0.480
Decrease	15.1 ± 38.7	6.3 ± 31.6	0.175	11.7 ± 27.0	14.0 ± 28.4	0.338
*p*-value i vs. f	0.184	0.006	-	<0.001	0.005	-

i at diagnosis; f after 3 months of treatment.

**Table 3:** Comparison in the total sample and by SIBO groups of the patients with normalization according to exhaled gases and clinical diagnosis.

Variable	H_2_-SIBO *n* = 56; *n* (%)	CH_4_-SIBO *n* = 123; *n* (%)	*p* * Value
Normalized gases			
Total G	19 (33.9)	55 (44.7)	0.174
CG	9 (37.5)	14 (40.0)	0.847
IG	10 (31.3)	41 (46.6)	0.133
*p*-value CG vs IG	0.625	0.507	
Normalized clinical manifestations			
Total G	40 (71.4)	90 (73.2)	0.808
CG	17 (70.8)	21 (60.0)	0.393
IG	23 (71.9)	69 (78.4)	0.454
*p*-value CG vs. IG	0.932	0.038	

* H_2_-SIBO vs. CH_4_-SIBO.

**Table 4:** Values at diagnosis of SIBO patients whose excreted gas values were normalized compared to those whose values were not normalized, according to SIBO groups.

Variable	Normalized H_2_-SIBO *n* = 19; 33.9%	*p*-Value N vs. NN	Normalized CH_4_-SIBO *n* = 55; 44.7%	*p*-Value N vs. NN
Age (years)	48.8 ± 14.1	0.092	48.1 ± 16.5	0.023
Women	14 (30.4)	0.236	47 (46.1)	0.503
BMI (kg/m^2^)	25.4 ± 5.0	0.402	23.4 ± 3.9	0.174
H_2_ (ppm)	37.6 ± 24.0	0.010	32.5 ± 38.5	0.101
CH_4_ (ppm)	6.5 ± 4.0	0.053	44.1 ± 26.2	0.004
Smokers	1 (20.0)	0.491	8 (50.0)	0.649
Chronically ill	5 (45.5)	0.395	11 (37.9)	0.401

N normalized values on the exhaled gas curve; NN not normalized.

## Data Availability

The original contributions presented in the study are included in the article, further inquiries can be directed to the corresponding author.
